# Negatively Charged MOF-Based Composite Anion Exchange Membrane with High Cation Selectivity and Permeability

**DOI:** 10.3390/membranes12060601

**Published:** 2022-06-10

**Authors:** Xiaohuan Li, Noor Ul Afsar, Xiaopeng Chen, Yifeng Wu, Yu Chen, Feng Shao, Jiaxian Song, Shuai Yao, Ru Xia, Jiasheng Qian, Bin Wu, Jibin Miao

**Affiliations:** 1 Key Laboratory of Environment-Friendly Polymeric Materials of Anhui Province, School of Chemistry & Chemical Engineering, Anhui University, Hefei 230601, China; c19201061@stu.ahu.edu.cn (X.L.); c20301112@stu.ahu.edu.cn (X.C.); c01914240@stu.ahu.edu.cn (Y.W.); c01914183@stu.ahu.edu.cn (Y.C.); c01914135@stu.ahu.edu.cn (F.S.); c01914255@stu.ahu.edu.cn (J.S.); c01914159@stu.ahu.edu.cn (S.Y.); 96047@ahu.edu.cn (R.X.); 89022@ahu.edu.cn (J.Q.); 2 Anhui Provincial Engineering Laboratory of Functional Membrane Materials and Technology, Department of Applied Chemistry, School of Chemistry and Materials Science, University of Science and Technology of China, Hefei 230026, China; noor@mail.ustc.edu.cn

**Keywords:** UiO-66, diffusive dialysis, cation separation, acid recovery

## Abstract

Every metal and metallurgical industry is associated with the generation of wastewater, influencing the living and non-living environment, which is alarming to environmentalists. The strict regulations about the dismissal of acid and metal into the environment and the increasing emphasis on the recycling/reuse of these effluents after proper remedy have focused the research community’s curiosity in developing distinctive approaches for the recovery of acid and metals from industrial wastewaters. This study reports the synthesis of UiO-66-(COOH)_2_ using dual ligand in water as a green solvent. Then, the prepared MOF nanoparticles were introduced into the DMAM quaternized QPPO matrix through a straightforward blending approach. Four defect-free UiO-66-(COOH)_2_/QPPO MMMs were prepared with four different MOF structures. The BET characterization of UiO-66-(COOH)_2_ nanoparticles with a highly crystalline structure and sub-nanometer pore size (~7 Å) was confirmed by XRD. Because of the introduction of MOF nanoparticles with an electrostatic interaction and pore size screening effect, a separation coefficient (S_HCl/FeCl2_) of 565 and U_HCl_ of 0.0089 m·h^−1^ for U-C(60)/QPPO were perceived when the loading dosage of the MOF content was 10 wt%. The obtained results showed that the prepared defect-free MOF membrane has broad prospects in acid recovery applications.

## 1. Introduction

A large amount of acidic wastewater comes from metallurgy [1], printing and dyeing [2], chemical production [3], mining of mineral resources [4], etc. In China, the pickling process of stainless steel in metallurgy alone produces at least 650,000 tons of acidic waste liquid every year [5]. The main hazards of acid mine drainage (AMD) to the environment are acid, sulfate, and metal ion pollution. Fe^2+^ is the most common metal ion in AMD, accounting for the vast majority of its content. Fe^2+^ and dissolved oxygen in the water will oxidize and precipitate into iron hydroxide, making the water brownish-yellow AMD have a low pH value and corrosion. It can dissolve metals in the soil, rocks, and sediments and increase the concentration of metal ions in the water environment [6]. Dumping these waste acids into the environment can corrode metal pipes and contaminate water and soil, posing a severe threat to human and animal health. Therefore, acid recovery from acidic waste liquid can save resources and play a significant role in protecting the environment. The emerging diffusion dialysis (DD) membrane separation technology driven by osmotic pressure has a high selectivity for acid recovery with no by-products, so it is cost-saving and more environmentally friendly. The development of effective and eco-friendly regeneration methods to recover acids from these industrial wastes has attracted the attention of many researchers and has excellent ecological and economic prospects. To improve the performance of the anion exchange membrane (AEM) in the DD process, Lin et al. [7] prepared a porous membrane using brominated polyphenylene ether (BPPO) with a high chemical stability and mechanical properties through non-solvent induced phase separation. They then performed one-step cross-linking and quaternization of porous BPPO membrane substrates with 1, 4-diazacyclic [2.2.2] octane. The acid dialysis coefficient was as high as 0.066 m·h^−1^, and the acid/salt separation coefficient was up to 96.9, which is more promising than the commercial AEM.

Metal-organic framework (MOF) is a new crystalline material characterized by a high porosity, elastic pore properties, ordered nanochannels, large functional groups, high thermal stability, and chemical stability [8]. Because of its simple synthesis, clear geometric structure, and excellent performance, MOF has been applied in many fields, such as energy recovery and storage [9], gas separation [10], catalysis [11], and sensors [12], and drug delivery [13]. Most MOF has a pore size distribution in the angstroms range, such as ZIF-8, MIL-101, and UiO-66, with a pore size of 6.0~8.0 A, and has been used in screening MOF-based films [14,15]. Large functional groups on the surface of MOF can make it charged, and MOF with functional groups can achieve the attraction/repulsion of target ions in water treatment. Therefore, when MOF is prepared into a membrane, H^+^ with a high activity, low charge, and small radius can quickly pass through the MOF membrane. At the same time, other metal cations (Fe^2+^, Zn^2+^, etc.) in the solution are trapped by electrostatic reactance and aperture screening due to the large hydration radius, so that the selective ion separation can be realized. Many MOF membrane separation works include the separation of single/multiple ions [16], seawater desalination [17,18], etc., but there are few studies on the application of MOF in acid recovery. UiO-66 is one of the most stable MOF in porous materials, which is used in gas separation [19], heavy metal ion separation [20,21], and dye adsorption [22,23,24,25] due to its stable pore size and framework structure. In addition, MOF nanoparticles are dispersed into the polymer matrix through mechanical agitation or ultrasonic methods, as well as the resulting MOF-based membrane. However, because of the different physical properties of the polymer matrix, high-load MOF particles tend to aggregate in the matrix [26,27], resulting in an uneven distribution of MOF in the membrane and interfacial voids, which further affects the selectivity of the membrane to the target ions. In long-term operation, these voids also reduce the mechanical strength of the membrane [28].

**Scheme 1:**
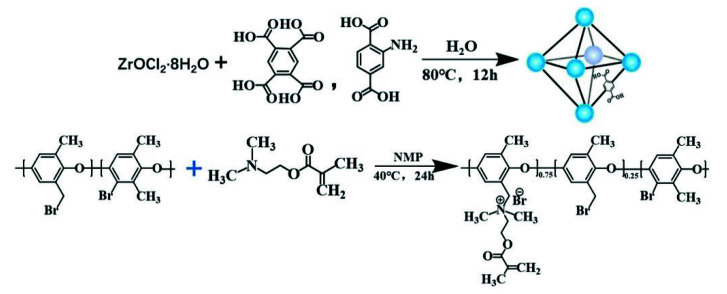
Preparation process of the UiO-66-(COOH)_2_ crystal and the synthesis of QPPO.

In this study, we designed UiO-66-(COOH)_2_ in a green solvent using the dual ligand method based on the background above. A series of defection-free mixed matrix membranes for four kinds of structures of MOF with different mass fractions were prepared by blending to realize the separation performance of HCl/FeCl_2_ (Scheme 1). In order to reduce the aggregation caused by the loading MOF, dimethylamine methacrylate (DMAM) was used for the quaternary ammonium reaction of BPPO. The -CO- in DMAM and -COOH in UiO-66-(COOH)_2_ formed hydrogen bonds, thus improving the dispersion of nanoparticles in the membrane. The design idea of the MOF membrane provides a broader platform for obtaining high-performance acid recovery membranes.

## 2. Materials and Methods

### 2.1. Materials

Zirconia octadeconium chloride (ZrOCl_2_·8H_2_O, AR, 99%), 2-amino-terephthalic acid (2-NH_2_-BDC, AR, 98.0%), benzenetetracarboxylic acid (H_4_BTEC, AR, 98.0%), sodium hydroxide (NaOH, AR, 96.0%), dimethylamine methacrylate (DMAM, 98%), and sodium chloride (NaCl, AR, 99.5%) were purchased from Aladdin Reagent. Acetic acid (CH_3_COOH, GR, 99.8%), chemical reagent of the Sinopharming Group; N-methylpyrrolidone (NMP, 99.73%), Shanghai Bidde Medical; ethanol (C_2_H_5_OH, AR, 99.7%), general purpose reagent; sodium sulfate, anhydrous (Na_2_SO_4_, AR, 99%), Shanghai McLean; hydrochloric acid (HCl, AR, 37%), Shanghai Test; ferrous chloride, tetrahydrate (FeCl_2_·4H_2_O, AR), Gansu Science; and brominated polyphenyl ether (BPPO, 42% bromination), lab made.

### 2.2. Synthesis of UiO-66-(COOH)_2_

UiO-66-(COOH)_2_ was prepared using the dual organic ligand [29,30,31,32,33]. The detailed process is as follows: 0.322 g (1 mmol) of ZrOCl_2_·8H_2_O was dissolved in 3 mL of water, then 1.23 mL of glacial acetic acid was added and heated at 60 °C for 2 h to form the metal precursor solution. Two organic ligands (2-NH_2_-BDC and H_4_BTEC) were added to 5 mL of deionized water in the proportions shown in Table 1. Then, 0.12 g (3 mmol) of NaOH was added, and it was heated at 60 °C for 10 min. Then, we allowed it to dissolve until it formed a brownish-red ligand solution, and cooled it to room temperature for later use. Finally, the ligand solution was added to the metal precursor solution to form a yellow solid. The mixture was heated at 80 °C for 12 h. The obtained product was washed with deionized water and ethanol three times and was centrifuged at 8000 RPM to remove the unreacted ligands and metal solution. The received UiO-66-(COOH)_2_ crystals were dried at 80 °C for 12 h to acquire a light-yellow solid powder. According to the different proportions of ligands, the four different types of UiO-66-(COOH)_2_ obtained were named U-C(50), U-C(60), U-C(70), and U-C(80).

### 2.3. Structure of UiO-66-(COOH)_2_/QPPO Membrane

#### 2.3.1. Preparation of QPPO Matrix

First, 10 g of BPPO was dissolved in 35 mL of NMP to make a transparent polymer solution. Then, a separate DMAM (3.467 g/30 mL of NMP) solution was prepared and added slowly to the BPPO solution (designated as QPPO) to avoid gelation for a quaternarization reaction.

#### 2.3.2. Preparation of UiO-66-(COOH)_2_/QPPO Mixed Matrix Membrane

The pre-ultrasonicated U-C(50) dispersion solutions, i.e., 0 wt%, 5 wt%, 7 wt%, 10 wt%, and 20 wt%/3.5 mL NMP, were added to the QPPO solution and stirred for 12 h. The well-dispersed MOF and QPPO solution was cast on a glass plate of 6 × 10 cm and was dried at 80 °C for 6 h. The obtain membrane (U-C(50)/QPPO) was immersed in deionized water for further analysis. The preparation methods of U-C(60)/QPPO, U-C(70)/QPPO, and U-C(80)/QPPO were the same as above.

### 2.4. Characterization

The X-ray diffraction (XRD) measurement was determined by SmartLab 9 KW, Rigaku Co., Ltd. (Tokyo, Japan). The BET was conducted using a fully automatic multi-station gas physics analyzer, ASAP 2460, McMortic Instruments LTD (Shanghai, China). Scanning electronic microscopy (SEM) J and electron microscopy (TEM) were carried out using a Regulus 8230, Hitachi, Japan, and field emission transmission electron microscope, Jeol, JEM-2100, Tokyo, Japan. The ICAPQ was examined using an inductively coupled plasma mass spectrometer, Thermo Fisher Technologies (Waltham, MA, USA). Diffusion dialysis (DD) unit device with an effective area of 4.9 cm^2^, which was self-made.

### 2.5. Water Uptake (WU) and Linear Elongation (LER)

WU and LER refer to the content of water and the membrane expansion (%). QPPO film with a size of 4 × 1 cm was dipped in deionized water for 24 h to make it fully absorb the water. The water attached to the film surface was removed using tissue paper, and we recorded the dimension changes of the wet film, which were denoted as W_wet_ and L_wet_, respectively. Then, the film was dried at 80 °C, and the quality and length of the dry film were recorded as W_dry_ and L_dry_, respectively. The WU and LER were calculated as follows:

(1) $$\mathrm{WU}=\frac{W_{\mathrm{wet}}-W_{\mathrm{dry}}}{W_{\mathrm{dry}}}\times 100\%$$

(2) $$\mathrm{LER}=\frac{L_{\mathrm{wet}}-L_{\mathrm{dry}}}{L_{\mathrm{dry}}}\times 100\%$$

### 2.6. Ion Exchange Capacity (IEC)

The IEC is generally expressed in terms of the number of exchange groups per gram of dry film (mmol/g). The QPPO membrane was dipped in a 1 mol/L NaCl solution for 24 h. The H^+^ in the membrane was completely exchanged with the Na^+^ in the solution. After that, the membrane was soaked in deionized water, and the fresh deionized water was replaced every 2 h. The process was repeated more than 10 times to completely clean the remaining NaCl solution on the membrane surface in order to obtain the chlorinated QPPO membrane. The membrane was dried at 80 °C and the dry membrane quality was recorded as W_dry_. Then, the membrane was soaked in the 100 mL 0.5 mol/L Na_2_SO_4_ solution for 8 h to release the Cl^−^. K_2_CrO_4_ and 0.1 mol/L AgNO_3_ were used as an indicator of the titrant for titration, respectively. The volume of AgNO_3_ solution was recorded as V_AgNO3_. The IEC calculation method was as follows:

(3) $$\mathrm{IEC}=\frac{V_{{\mathrm{AgNO}}_{3}}\times 0.1}{W_{\mathrm{dry}}}$$

### 2.7. Ion Separation Performance Test

The diffusion dialysis device consisted of two compartments, a feeding and a diffusion chamber, and the membrane was fixed in the middle of the two compartments. The effective area of the membrane was 4.9 cm^2^. The membrane was first immersed in a simulated waste acid solution (1 mol·L^−1^ HCl and 0.2 mol·L^−1^ FeCl_2_) for 12 h, and was then thoroughly cleaned with deionized water to remove the residual acid on the surface before DD. During the experiment, 40 mL of feed solution and 40 mL of deionized water were injected into the feeding chamber and diffusion chamber, respectively, and the mixture was stirred to eliminate the concentration polarization. Each film was tested for 1 h at 25 °C. The concentration of H^+^ was titrated with 0.01 mol·L^−1^ NaOH solution using methyl red and bromocresol green as a mixed indicator (V methyl red/V bromocresol green ratio of 1:3), and the solution changed from red to blue-green, which was the endpoint of the titration. At the same time, the concentration of Fe^2+^ was determined using an inductively coupled plasma mass spectrometer (ICP, ICAPQ, Thermo Fisher Technologies, Waltham, MA, USA). The diffusion coefficients (U) of HCl and FeCl_2_ can be calculated using the following equation:

(4) $$U=\frac{M}{S\cdot t\cdot \Delta C}$$

where M represents the mole amount of diffusion of a single component (HCl or FeCl_2_), S represents the effective membrane area, and t represents the diffusion time. C represents the logarithmic mean of the concentration difference between two chambers, as defined in the following formula:

(5) $$\Delta C=\;\frac{C_{f}^{0}-C_{d}^{t}-C_{f}^{t}}{\ln\left[\left(C_{f}^{0}-C_{d}^{t}\right)/C_{f}^{t}\right]}$$

where C^0^ f and C^t^ f are the concentration of a single component (HCl or FeCl_2_) in the feed at time 0 and t, respectively. C^t^_d_ represents the concentration of a single component (HCl or FeCl_2_) in the dialysate at time t.

The ratio of diffusion coefficients of HCl (U_HCl_) and FeCl_2_ (U_FeCl2_) is the separation factor (S):

(6) $$S=\;\frac{U_{\mathrm{HCl}}}{U_{{\mathrm{FeCl}}_{2}}}$$

## 3. Results and Discussion

### 3.1. Preparation and Characterization of UiO-66-(COOH)_2_ Crystal

**Figure 1:**
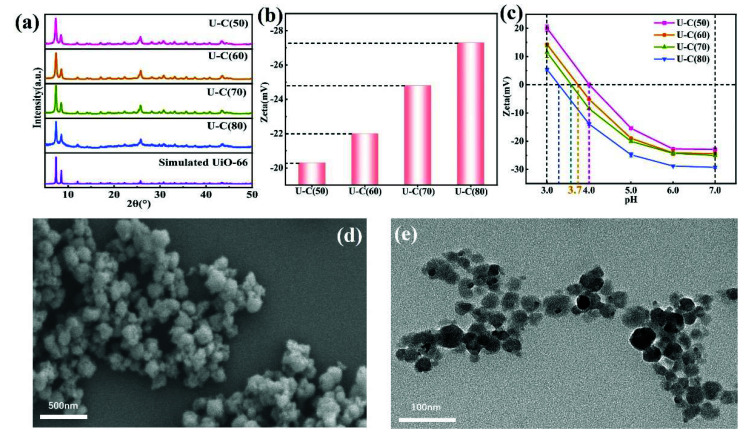
(**a**) XRD patterns of four different carboxyl groups of UiO-66-(COOH)_2_ nanoparticles and simulated UiO-66. (**b**) Zeta potential of four different carboxyl groups of UiO-66-(COOH)_2_ nanoparticles. (**c**) Zeta potential of UiO-66-(COOH)_2_ with different H_4_BTEC contents as a function of pH. (**d**) SEM and (**e**) TEM images of the U-C(50) nanoparticles.

The X-ray diffraction spectra show the crystal structure of the MOF nanoparticles. As can be seen from Figure 1a, the synthesized UiO-66-(COOH)_2_ shows intense diffraction peaks at 2θ = 7.4°, 8.5°, 14.8°, 17.1°, 25.8°, and 30.8°. The peaks at the six locations correspond to the crystal planes of (111), (200), (222), (400), (442), and (711). Compared with the XRD of the simulated UiO-66, there is no visible peak shift, which confirms the highly crystalline structure of the MOF nanoparticles. In addition, compared with the XRD of the standard UiO-66, the diffraction peaks of four kinds of UIO-66-(COOH)_2_ were diffused and widened. According to the Debye-Scherrer formula [34], this is as a result of the smaller particle size of the MOF nanoparticles (~50 nm), which is consistent with the results of the transmission electron microscopy.

(7) $$D=\frac{K\gamma }{\mathrm{Bcos}\theta }$$

Sherrer’s formula describes the relationship between grain size and half peak width of the diffraction peak. K is Scherrer’s constant, K = 0.89; D is the average thickness of grain perpendicular to the grain plane (A); B is the half-height width of the diffraction peak of the measured sample, i.e., the half-peak width; and θ is the Bragg diffraction angle, and the unit is Angle (°). γ is the wavelength of the X-ray, which is generally 1.54056 Å

The zeta potential results characterize the degree of charge of nanoparticles. It can be seen from Figure 1b that the Zeta potentials of the synthesized nanoparticles are all negative, indicating that the surface of the particles is negatively charged, which proves that UiO-66-(COOH)_2_ was successfully synthesized using the dual ligand method. In addition, with the increase of the proportion of in the organic ligand, the Zeta potential decreases from −20.3 mV to −27.3 mV, and the electrostatic repulsion between the particles increases gradually, indicating that the proportion of -COOH in the nanoparticles presents a trend of gradual increase. The electrostatic interaction between cation and the UiO-66-(COOH)_2_ was modulated by varying the pH of electrolyte solution. Due to the existence of carboxyl groups (-COOH) on the UiO-66-(COOH)_2_ framework, the charge properties of the channel wall are dependent on the pH of ionic solutions. We measured the Zeta potential of UiO-66-(COOH)_2_ with different from pH 3.0 to 7.0 of FeCl_2_ solution and obtained the isoelectric point at ≈3.2~4.0, which means the channels of are neutral when the pH is around 3.2~4.0 with different H_4_BTEC content (Figure 1c). Consequently, the channels of UiO-66-(COOH)_2_ were positively charged when the pH is below 3.2~4.0, while the channels of UiO-66-(COOH)_2_ were negatively charged when the pH is above 3.5~4.0. The morphology of UiO-66-(COOH)_2_ nanoparticles was characterized by SEM and TEM. As can be seen from Figure 1d,e, the size of MOF nanoparticles is around 50 nm, which also explains the diffraction peak broadening in XRD characterization.

**Figure 2:**
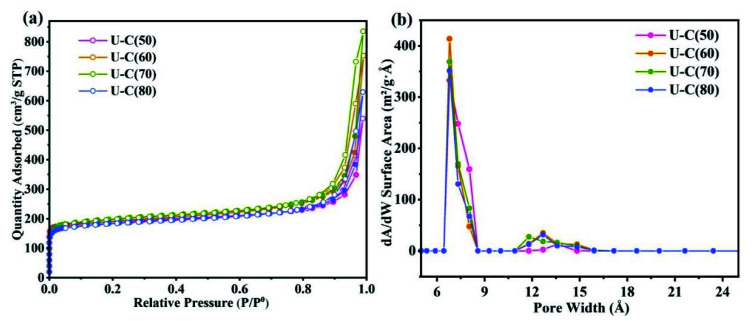
(**a**) N_2_ adsorption isotherm and (**b**) pore size distribution of four different composition of ratio of ligand UiO-66-(COOH)_2_ nanoparticles.

As can be seen from the Figure 2a, N_2_ adsorption capacity increased with the increase of H_4_BTEC content for U-C(50) (544.4 m^2^·g^−1^), U-C(60) (596.7 m^2^·g^−1^), and U-C(70) (617.9 m^2^·g^−1^). However, due to the steric hindrance caused by the high content of carboxylic acid, the gas adsorption process was affected and the nitrogen adsorption capacity decreases of U-C(80) decreased to 570.3 m^2^·g^−1^. It can be seen from the pore size distribution diagram that the pore size of the four UiO-66-(COOH)_2_ nanoparticles are all at 7 Å, and the pore size distribution at 11–16 Å is caused by the stacking of nanoparticles (Figure 2b). In the process of acid recovery, H^+^ with a small radius can quickly pass through the MOF membrane and increase the diffusion rate of acid, while Fe^2+^ is trapped by the MOF membrane due to the large hydration diameter, thus improving the selective separation performance of H^+^/Fe^2+^.

**Figure 3:**
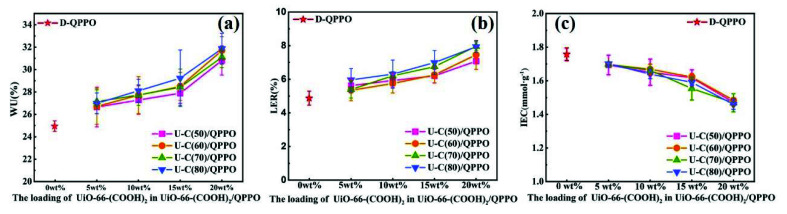
(**a**) WU, (**b**) LER, and (**c**) IEC of UiO-66-(COOH)_2_/QPPO membranes with four kinds of MOF of different carboxyl group contents under different MOF fillers.

Figure 3a,b shows WU and LER of UiO-66-(COOH)_2_/QPPO with different filler loading. The WU of blank QPPO (D-QPPO) was 25%, and the water content increased with the increase of filler content. In addition, with the increase of H_4_BTEC content, the water content also increased when the filler amount remained unchanged. This is because the carboxyl group is a hydrophilic group. When the carboxyl content in the system increases, the hydrophilicity of the film increases, and then the water content increases, up to 31.7% (U-C(80)/QPPO-4). This result is also reflected in the change of the linear elongation of the membrane. After the membrane absorbs water and expands, the linear elongation increases from 4.9% (D-QPPO) to 7.9% (U-C(80)/D-QPPO). Figure 3c shows the IEC of UiO-66-(COOH)_2_/QPPO films with different filler loading. The IEC value of D-QPPO was 1.759 mmol/g. With the increase of filler loading, the IEC value of UiO-66-(COOH)_2_/QPPO gradually decreased, due to the stronger electrostatic interaction between the carboxyl group in U and the quaternary ammonium group in QPPO. However, such IEC values can still meet the requirements of ion exchange.

### 3.2. H^+^/Fe^2+^ Separation Performance of UiO-66-(COOH)_2_/QPPO Membrane

**Figure 4:**
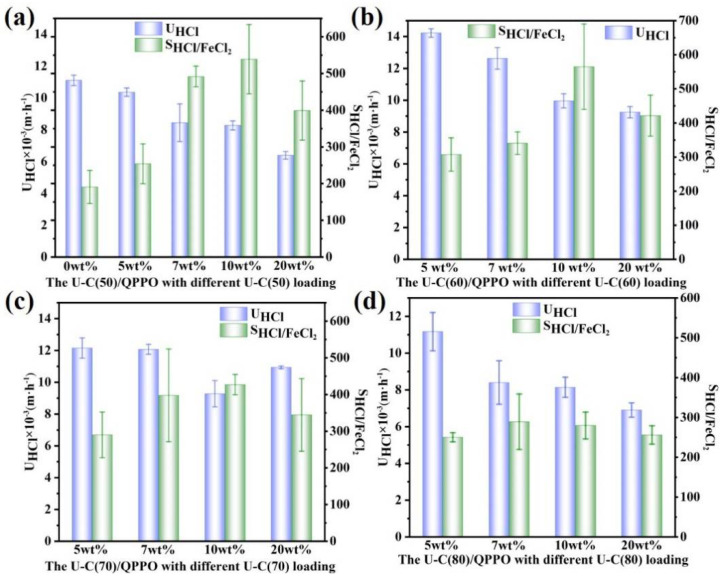
H^+^ dialysis coefficient (U_HCl_) and H^+^/Fe^2+^ separation factor (S_HCl/FeCl2_) of UiO-66-(COOH)_2_/QPPO with four kinds of MOF with different carboxyl group contents under different MOF loadings. (**a**) U-C(50)/QPPO, (**b**) U-C(60)/QPPO, (**c**) U-C(70)/QPPO, and (**d**) U-C(80)/QPPO.

In order to characterize the application of UiO-66-(COOH)_2_/QPPO in acid recovery, DD experiments were carried out using HCl/FeCl_2_ as a mixed feed solution. Figure 4 shows the acid recovery results of UiO-66-(COOH)_2_/QPPO with different carboxyl contents of MOF and different packing amounts. The separation factor of HCl/FeCl_2_ was relatively low (S = 191) because the D-QPPO membrane did not contain UiO-66-(COOH)_2_ with a pore size screening function that could intercept Fe^2+^. With the increase in UiO-66-(COOH)_2_ loading, the ability of UiO-66-(COOH)_2_/QPPO to separate ions gradually increased, and the separation factor of HCl/FeCl_2_ was up to 565 (U-C(60)/QPPO, 10 wt%). When the filler loading increased to 20 wt%, the separation factors of the four UiO-66-(COOH)_2_/QPPO membranes decreased, because UiO-66-(COOH)_2_ with a high loading would aggregate in the membrane matrix, resulting in pore defects of the membrane, and Fe^2+^ diffused through the membrane pores to the permeable side, resulting in a reduced separation performance, which could be proven by the SEM results in the previous paper. The higher the load, the more obvious the agglomeration phenomenon, and the more serious the membrane defects.

In addition, the diffusion coefficient of H^+^ in UiO-66-(COOH)_2_/QPPO with the same carboxyl group content decreased gradually with the increase in UiO-66-(COOH)_2_ additions. This is because when UiO-66-(COOH)_2_ increased, the content of carboxyl groups in the membrane also increased. Under the action of electrostatic repulsion, the diffusion rate of Cl^−^ to the permeable side slowed down. According to the principle of electric neutrality, the transmission rate of H^+^ decreased under the influence of Cl^−^. However, because of the presence of carboxyl groups, the proton transport path was constructed. When the carboxyl group content in MOF continued to increase, the H^+^ diffusion coefficient increased, as well as the H^+^ diffusion coefficient of U-C(60)/QPPO.

**Figure 5:**
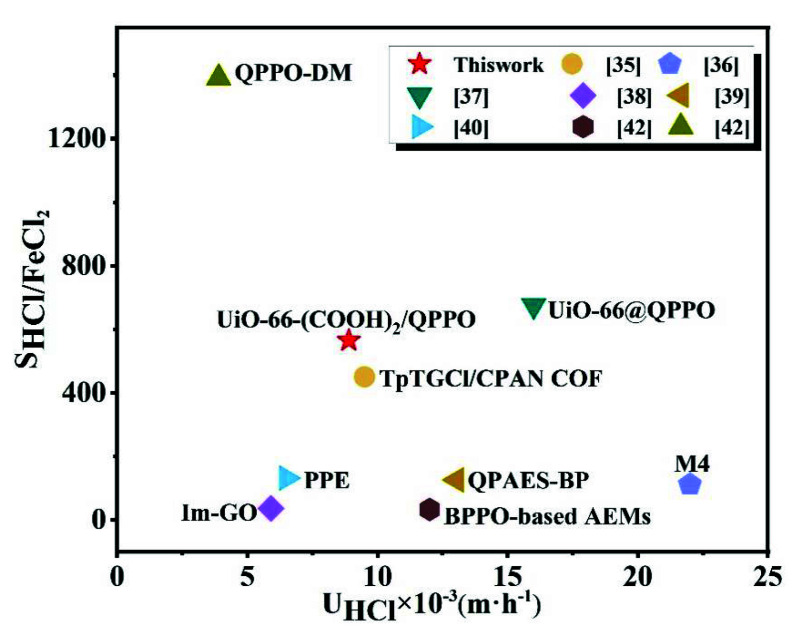
S_HCl/FeCl2_ versus U_HCl_ of the reported membranes under diffusion dialysis [35,36,37,38,39,40,41,42].

Specifically, U-C(60)/QPPO membranes exhibited selectivity for H^+^/Fe^2+^ of ≈565, while maintaining a high monovalent cation permeation rate of 0.0089 m·h^−1^, much higher than most those of the previously reported membranes (Figure 5). Such an excellent performance resulted from the interaction of carboxyl groups on the frame structure with different ions.

### 3.3. Structure and Characterization of U-C(6)/QPPO Membrane

**Figure 6:**
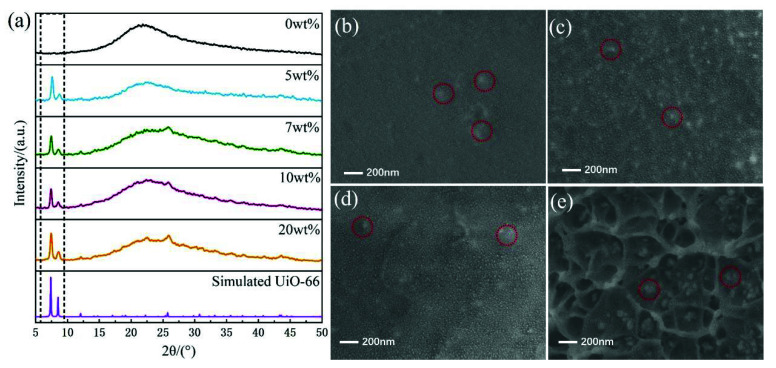
(**a**) XRD patterns of U-C(60)/QPPO with different MOF fillers. (**b**–**e**) SEM images of U-C(60)/QPPO with different filler loading. ((**b**). 5 wt%; (**c**). 7 wt%; (**d**). 10 wt%; (**e**). 20 wt%). U-C(60) in U-C(60)/QPPO was marked by red circle.

Because of the high separability of U-C(60)/QPPO, the membrane structure of U-C(60)/QPPO was further characterized. As can be seen from Figure 6a for the XRD patterns of U-C(60)/QPPO with different filler loadings, when the filler content was greater than 7 wt%, the XRD diffraction peaks of UiO-66 showed characteristic peaks at 2θ = 7.4°, 8.5°, and 25.8°, indicating that the crystal structure of UiO-66-(COOH)_2_ mixed with QPPO did not change significantly, and the structure was relatively intact. This is beneficial for the separation of H^+^/Fe^2+^. However, when the filler amount was 5 wt%, no characteristic diffraction peak appeared at 25.8° in the XRD pattern, because the filler amount was too low, and the filler was covered by the film matrix QPPO, and thus no characteristic peak was displayed.

Figure 6b–e shows the SEM of the sections of U-C(60)/QPPO with different filler loadings. The presence of U-C(60) can be observed in the cross-section of the membrane (red circle area), and the number of U-C(60) significantly increased with the increase in the filler amount. When the filler content was 5 wt% and 7 wt%, U-C(60) was evenly dispersed in the membrane, and the cross-section structure of the membrane was uniform and continuous, without obvious membrane gaps. However, after the addition of 10 wt% UiO-66-(COOH)_2_, the cross-section roughness of U-C(60)/QPPO increased slightly and U-C(60) appeared to have slight agglomeration. With the addition of U-C(60) up to 20 wt%, the agglomeration of MOF in the membrane was more obvious. The SEM shows that the cross-section of the membrane was honeycomb, and its roughness increased significantly, which led to an increase in the gap inside the membrane, resulting in structural defects and in the destruction of the proton transport path. In the process of acid recovery, Fe^2+^ could enter the permeable side through the membrane defect, which eventually reduced the separation performance of H^+^/Fe^2+^.

**Figure 7:**
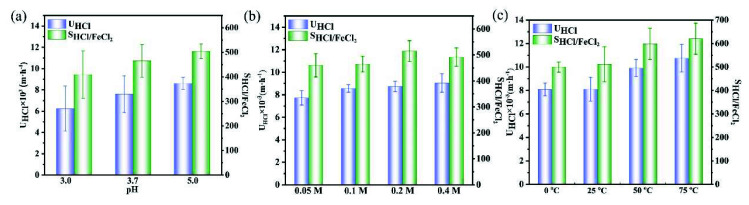
(**a**) H^+^ dialysis coefficient (U_HCl_) and H^+^/Fe^2+^ separation factor (S_HCl/FeCl2_) of U-C(60)/QPPO membranes with different pH values. (**b**) H^+^ dialysis coefficient (U_HCl_) and H^+^/Fe^2+^ separation factor (S_HCl/FeCl2_) of U-C(60)/QPPO membranes with different ionic strengths. (**c**) H^+^ dialysis coefficient (U_HCl_) and H^+^/Fe^2+^ separation factor (S_HCl/FeCl2_) of U-C(60)/QPPO membranes with different temperatures.

We chose three representative pH values to study the U_HCl_ and S_HCl/FeCl2_ with U-C(60)/D-QPPO, that is, 3.0, 3.7, and 5.0, corresponding to the positively, neutrally, and negatively charged channel walls, respectively (Figure 7a). The U_HCl_ and S_HCl/FeCl2_ increased gradually with the increase in pH value from 3.0 to 5.0. This is because the positively charged channels imposed an electrostatic repulsion toward cations at a lower pH, hindering the transport of H^+^, and the negatively charged channels showed an electrostatic attraction toward cations at a higher pH, facilitating the transport of H^+^. However, the permeation rate of Fe^2+^ was extremely low and hardly changeable to the varied pH, which indicated that there was a strong electrostatic adsorption between the carboxyl group on U-C(60) and Fe^2+^.

In order to analyze the influence of the separation system on the separation effect, the ionic strength and the temperature were investigated. As shown in Figure 7b, with the increase in ionic strength, U_HCl_ presented an upward trend, while S_HCl/FeCl2_ first increased and then decreased. The reason for this result is that the increase in ionic strength led to the rapid movement of a large number of protons under the promotion of U-C(60), thus obtaining a high U_HCl_ value. However, the increase in Fe^2+^ enhanced the enhancement of the electrostatic interaction with the -COO- in U-C(60), which reduced the effect of H^+^ promotion to a certain extent, thus affecting S_HCl/FeCl2_. The temperature of the system is another important factor affecting U_HCl_ and S_HCl/FeCl2_. As can be seen from Figure 7c, with the rise in temperature, both U and S tended to increase. The reason for obtaining this result was that the ion migration ability was improved with the rise in temperature, and the higher U_HCl_ and S_HCl/FeCl2_ were reflected under the promotion of U-C(60).

## 4. Conclusions

A series membrane with different UiO-66-(COOH)_2_ fillers was constructed using the blending method. UiO-66-(COOH)_2_ with a free carboxylic acid structure plays an important role in the membrane performance. The acid recovery performance was investigated with DD using HCl/FeCl_2_ as the simulated waste acid. With the increase in the carboxyl group content in UiO-66-(COOH)_2_, the diffusion coefficient U_HCl_ decreased gradually, and the S_HCl/FeCl2_ increased first and then decreased. When the U-C(60) content was 10 wt%, the S_HCl/FeCl2_ and U_HCl_ of U-C(60)/QPPO reached 565 and 0.0089 m·h^−1^, respectively. This is as a result of the electrostatic interaction between the free carboxyl group in U-C(60) and Fe^2+^, which promotes the efficient transmission of H^+^. This MOF-based membrane has broad application prospects in the acid recovery field.

## Figures and Tables

**Table 1:** Ligand ratios for the preparation of four kinds of UiO-66-(COOH)_2_.

Ligands	U-C(50)	U-C(60)	U-C(70)	U-C(80)
2-NH_2_-BDC	0.322 g	0.322 g	0.322 g	0.322 g
H_4_BTEC	0.091 g	0.072 g	0.054 g	0.036 g

## Data Availability

Data are available on request from corresponding authors.
